# Three-Dimensional Gravity Inversion Based on Attention Feature Fusion

**DOI:** 10.3390/s24175697

**Published:** 2024-09-01

**Authors:** Chen Chen, Houpu Li, Yujie Zhang, Xiaomei Jin, Jianfeng Liu

**Affiliations:** 1 School of Mathematics and physics, China University of Geosciences, Wuhan 430074, China; 2 College of Electrical Engineering, Naval University of Engineering, Wuhan 430074, China

**Keywords:** gravity anomalies and earth structure, inverse theory, neural networks, attention feature fusion

## Abstract

Three-dimensional gravity inversion is a process of obtaining the location, shape, and physical property parameters of underground anomaly sources using gravity anomaly data observed on the surface. In recent years, with the rapid development of data-driven methods, the application of deep learning (DL) to 3D gravity inversion has also attracted wide attention and achieved certain results. In this paper, based on the U-Net network, a three-dimensional gravity inversion method using an attention feature fusion mechanism is proposed. Using U-Net as the basic framework, the coarse-grained semantic features and fine-grained semantic features in the encoder and decoder are connected by long hops, and the global and local semantic features are aggregated through the attention feature fusion module, which avoids feature loss in the network training process. Compared with the inversion results of the U-Net network, the proposed method has a higher vertical resolution and effectively alleviates the influence of the skin effect on three-dimensional gravity inversion. Ablation experiments show that the attention feature fusion module is the key to improving the vertical resolution and prediction accuracy of inversion results. Noise experiments show that the inversion network in this study has a strong anti-noise ability and good generalization performance. The experimental results of the inversion network used in the prediction of the SAN Nicolas deposit in Mexico show that the inversion network can clearly predict the basic location and general shape of the sulfur deposit, and the results are in good agreement with the known geological data.

## 1. Introduction

Gravity exploration is a method to calculate the location and physical property parameters of a geological body through the density difference between the underground geological body and surrounding rocks [1]. With the development of space exploration technology, satellite gravity, airborne gravity, and microgravity measurements have been widely applied [2]. In the field of gravity exploration, the research hotspot of quantitative interpretation of gravity anomaly data is three-dimensional gravity physical property inversion, which is mainly used for the exploration of geological structures and salt domes and the delineation of coal basins in oil and gas prospect areas. This study uses deep and regional geological structures to predict crustal movements and cooperates with other geophysical exploration methods to search for metallic and non-metallic minerals [3,4].

Traditional geophysical inversion often adopts linear inversion. The linear inversion theory was first proposed by Backus and Gilbert [5] and later applied to geophysical inversion by Parker [6]. In order to solve the problem of multiple solutions in linear inversion and improve the stability of solutions, Tikhononv and Arsenin [7] introduced regularization technology into linear inversion. However, the final result of the linear iterative inversion method depends on the selection of the initial model to a large extent, so the linear inversion method is often limited in practical application. The proposed nonlinear inversion method solves the problem that the objective function of linear inversion is easy to fall into the local minimum value to a large extent. Common nonlinear inversion methods include the genetic algorithm [8], simulated annealing algorithm [9], ant colony algorithm [10], global particle swarm algorithm [11], Hunger Games Search optimization [12], and neural network algorithm [13]. Essa describes a fast-imaging technique, the “R-parameter imaging technique”, for the interpretation of gravity data measured along profile [14]. Nonlinear inversion has better optimization ability and inversion efficiency, especially the deep learning method.

Due to the breakthrough application of the deep neural network (DNN) in speech recognition and image recognition [15], the DNN has been widely used in a variety of scenarios and has become the basic network for the practical application of artificial intelligence technology [16,17,18,19,20,21].

In recent years, Huang et al. [22,23] constructed simulation data using the random walk method and used the V-Net network for large-scale gravity inversion. Yang et al. [24] used an improved U-Net system for three-dimensional gravity inversion. Li et al. [25] developed GV Net using convolutional neural networks for inverting residual gravity anomalies. Xu et al. [26] proposed a fast reconstruction method for underground density models based on ResUnet. Lv et al. [27] introduced a multi-task approach to accurately locate geological bodies first and then performed precise inversions on them. Wang et al. [28] proposed using multi-scale functional MS-UNet networks to alleviate the problem. Zhang et al. proposed a novel 3D gravity inversion method based on encoder–decoder neural networks [29]. The successful application of these methods indicates that as a technology with adaptive learning ability and nonlinear mapping ability, the deep learning method may become a supplement or substitute for traditional methods in the future.

Although the current gravity inversion technology is relatively mature in development, due to the inherent properties of the geophysical field, there are still many problems unsolved in the process of solving, such as the skin effect. Since the shallow model unit makes a greater contribution to the observation surface than the deep model unit, when the test set is tested with the trained neural network, a clearer outline is often shown for shallow units, while the outline is more blurred for deep units. Although U-Net is an effective means to carry out three-dimensional gravity inversion, it still has some limitations. The inversion results have a poor effect in the deep part of the model and a low resolution in the vertical direction.

In order to solve the above problems, this paper refers to the framework of U-Net and uses residual network and attention mechanism to build a neural network containing an attention feature fusion module to improve the accuracy of three-dimensional gravity inversion in the vertical direction.

The rest of the research is organized as follows. In Section 2, the basic principles of gravity forward modeling and gravity inversion are briefly introduced. In Section 3, the structure of the inversion network designed in this paper is introduced in detail, including the residual module, the attention feature fusion module, and the multi-scale channel attention module. Section 4 introduces the dataset, implementation details, and experimental results of the experiment. The noise experiment, contrast experiment, and ablation experiment are designed and carried out, and the inversion network is applied to the processing of real data. The conclusions are contained in Section 5.

## 2. Background

The purpose of gravity forward is to understand gravity anomalies caused by geologic bodies of different sizes, occurrences, and densities. Gravity inversion is used to obtain the density, shape, and other physical property parameters of the geological body caused by the anomaly. In the current methods of solving inversion problems, forward modeling is an important part of the inversion process.

### 2.1. Forward Modeling

In the calculation of arbitrary shape, size, and geologic body caused by the gravity anomaly, on the surface of the observation area, plasmid profile control is usually divided into countless volume elements, assuming a split unit for location (*ξ*, *η*, *ζ*) and the density of *ρ* (*ξ*, *η*, *ζ*). The gravity field generated by the body at the measuring point *P*(*x*, *y*, *z*) can be expressed as follows [30]:

(1) $$F=\gamma {∭}_{V}\rho (\xi ,\eta ,\zeta )\frac{1}{r^{3}}dv,$$

where *V* represents the volume of the geological body, *r* represents the distance from the volume element *Q*(*ξ*, *η*, *ζ*) to the observation point *P*, and *γ* represents the gravitational constant. Then, the gravity anomaly of the body is the component of the gravity field in the vertical direction, which can be expressed as follows:

(2) $$g=\frac{\partial V}{\partial z}=\gamma {∭}_{V}\rho (\xi ,\eta ,\zeta )\frac{z-\zeta }{r^{3}}dv.$$

**Figure 1:**
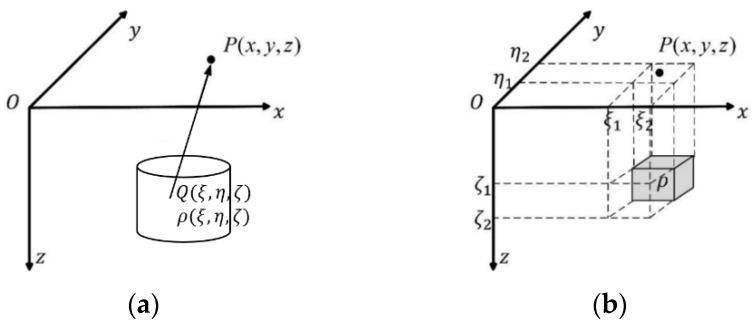
Schematic diagram of gravity anomaly calculation: (**a**) arbitrary geological body, (**b**) cuboid model.

In the actual forward modeling of gravity anomalies, the underground abnormal body is usually divided into several cuboid units, as shown in Figure 1b, and then the anomalies generated by each cuboid unit on the measuring point are calculated. The sum of the anomalies generated by all cuboid units on the measuring point is the anomalies generated by the whole model body in the underground half space on the observation point. When the number of units divided is large enough and the volume of units is small enough, the above method can fit the geological body of any shape. Then, the approximate calculation formula of gravity anomaly can be expressed as follows:

(3) d=Gm,

where ***d*** is a p×1 vector composed of gravitational outliers at each observation point; ***m*** is a q×1 vector composed of the density of each element after the partition of the geological body; and ***G*** is the sensitivity matrix, and the matrix elements in $g_{ij}$ are a quantitative description of the first *j* a model unit for the first *i* observation point the contribution of gravity [31].

### 2.2. Inversion Modeling

The gravity inversion problem can be understood as the inverse process of the forward modeling problem, where ***m*** in Equation (3) is unknown, and the density parameter ***m*** of the geologic body in the model space should be deduced by the known observation data ***d***. However, at this time, we are faced with a problem. Since the number of observation points is far less than the number of geologic body sections, that is, the dimension of ***d*** is less than that of ***m***, solving the gravity inversion problem solves the underdetermined equations, in which case the solution of the equations is non-unique, and the solution is obviously unstable. In this case, the vertical resolution of the inversion result will be greatly reduced, and the skin effect will appear.

The least square method is often used to fit the objective function in inversion modeling, which can be expressed as follows:

(4) $$\phi ={\left\|d-Gm\right\|}^{2}.$$

In order to reduce the impact of measurement errors and multi-solution problems, Tikhonov proposed regularization inversion [32], adding constraints to the objective function to limit the scope of the model. The objective function can be rewritten as follows:

(5) $$\phi =\phi_{d}+\alpha \phi_{m}{=\left\|d-Gm\right\|}^{2}+\alpha {\left\|m-m_{0}\right\|}^{2},$$

where $\phi_{d}$ denotes data fitting item, $\phi_{m}$ represents a model-fitting item, and *α* represents a regularization parameter. $m_{0}$ indicates the initial model and is associated with prior information; when the prior information is insufficient or absent, let $m_{0}$ be 0. What is introduced here is the regularization function under the two-norm constraint. In the study of practical problems, there are many forms of regularization functions [7].

This paper focuses on the DL data-driven inversion method, which deduces the density model directly from the observed gravity anomaly data and depicts the underground anomaly with sharp boundaries. The DNN represents the mapping relationship from the data domain to the model domain through a complex, nonlinear network [28], which can be expressed as follows:

(6) m=F(d).

Unlike the regularization inversion proposed by Tikhonov, which minimizes the error between the predicted outlier and the observed outlier, DNN inversion minimizes the error between the predicted density model and the theoretical density model.

In the field of gravity inversion based on DL, Huang et al. [23] used the U-Net network to perform sparse gravity inversion. The experimental results show that the U-Net network can better reflect the location, shape, and boundary of underground geological bodies, but the influence of the skin effect still exists.

Li et al. [33] advocate feature fusion after the skip connection from the perspective of attention. Inspired by Li, this paper uses the attention feature fusion mechanism to fuse the encoded feature and the decoded feature. On this basis, a three-dimensional inversion network based on the attention feature fusion mechanism is proposed, which enables the network to learn the physical property information of the deep part of the underground model well and improves the reconstruction accuracy of the deep part. In this way, the skin effect problem in three-dimensional gravity inversion can be alleviated. This paper constructs the encoder and decoder by stacking residual modules, and the splicing of features between the encoder and decoder will be realized using the attention fusion mechanism. The training of the inversion network requires a large amount of data, but the data sample type used by most methods is relatively single, and the shape of the anomaly source is relatively regular, resulting in poor generalization of the inversion network. In order to improve the generalization of the network, on the basis of constructing the conventional model, this paper adds a random model composed of random walk mode to the dataset to increase the randomness and diversity of the training data. The method proposed in this paper can better reverse the physical property information of the deep model and alleviate the influence of the skin effect to a certain extent. At the same time, the noise test and real data experiment show that the proposed method can effectively improve the generalization of the inversion network and obtain a clear and focused anomaly source, which provides a basis for practical application.

## 3. Methodology

The encoder–decoder structure of U-Net excels in extracting intricate two-dimensional characteristics from gravity anomaly data, facilitating the reconstruction of a precise three-dimensional density model. Nevertheless, the simplistic combination of encoded and decoded features within U-Net often leads to the erosion of vital feature information, consequently hindering the prediction model’s capability to accurately capture nuanced, in-depth details. Therefore, this paper refers to the framework of U-Net and uses residual network and attention mechanism to build a neural network containing an attention feature fusion module to improve the accuracy of three-dimensional gravity inversion in the vertical direction.

This paper designs an inversion network based on U-Net structure and, combined with an attention feature fusion module, aims at making the network fully learn the global and local features in the training process so as to alleviate the influence of the skin effect to a greater extent. A density model dataset named GravInv is constructed, which consists of seven types of models. Through experiments, the inversion network we designed has a better vertical resolution on both synthetic data and actual data. The design comparison experiment and ablation experiment verify that adding the attention feature fusion module to the neural network is the key to improving the resolution and inversion accuracy of the deeper model, and the skin effect is alleviated to a large extent.

### 3.1. Construction of the Inversion Network

**Figure 2:**
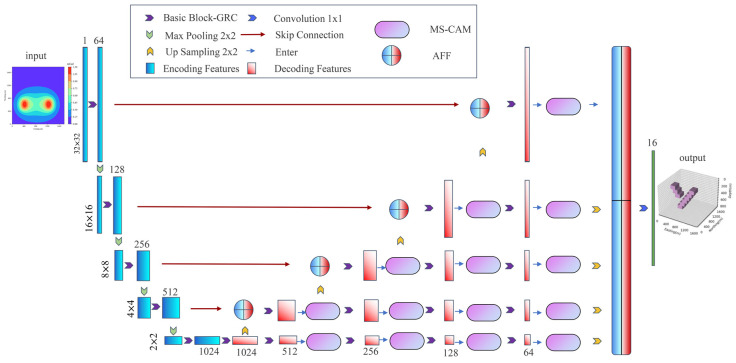
Inversion network structure diagram.

In order to make the gravity three-dimensional inversion task better reconstruct the depth part density model, high-resolution inversion is achieved in the vertical direction. In this paper, our inversion network is based on the framework of U-Net [34], which includes a contraction path for capturing context and a symmetric expansion path for supporting accurate localization, and the convolution layer is replaced by Basic Block [35] for training a deeper network. Replace the simple feature matching process between the encoder and decoder with the attention feature fusion (AFF) module [36], which uses the idea of the attention mechanism for feature fusion, and then extract the features again. In order to obtain better feature representation, which can help improve the resolution of the prediction results, the multi-scale channel attention module (MS—CAM) [36] will further polymerize the global and local features and finally extract the features of different scales of the characteristics of fusion. The network structure is shown in Figure 2.

The encoder on the left uses Basic Block to extract features from the gravity anomaly observation data and then changes the size of the feature map through the maximum pooling layer. It is worth mentioning that after the convolutional layer in each Basic Block, there is a BN layer and a ReLU activation function layer to prevent gradient disappearance or gradient explosion problems.

**Figure 3:**
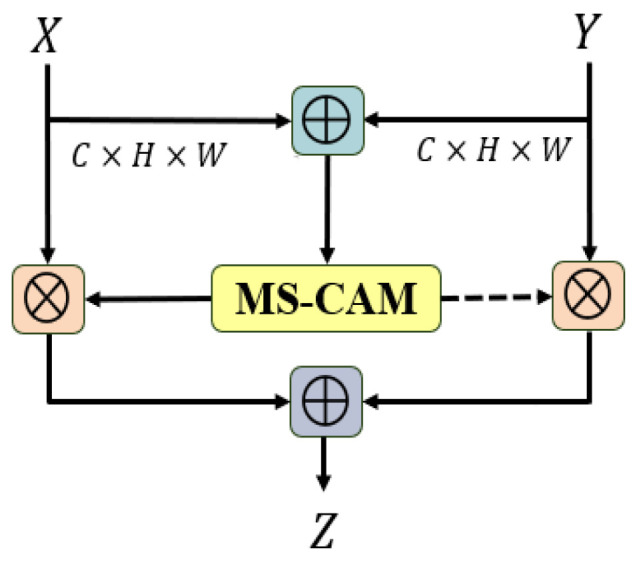
AFF structure.

The decoder on the right upsamples the gravity anomaly features extracted by the encoder to the same size as the features of the previous layer. Then, the features extracted by the encoder and the features sampled by the decoder are fused in AFF through the long-skip connection. The features after fusion are further extracted by Basic Block. AFF can be expressed as follows:

(7) $$Z=M\left(X⨄Y\right)⊗X+(1-M\left(X⨄Y\right))⊗Y,$$

where $Z\in R^{C\times H\times W}$ is the fusion feature with the number of channels *C* and size *H* × *W* and ⨄ indicates the initial feature integration. The AFF module [36] is shown in Figure 3. After the initial feature integration, features X and Y obtain the attention fusion weights through the MS-CAM. The fusion feature Z is obtained by adding the initial features *X* and *Y* to the inner product of the corresponding weights, respectively. In the AFF module, for simplicity, we chose the addition of feature elements as the initial feature integration.

**Figure 4:**
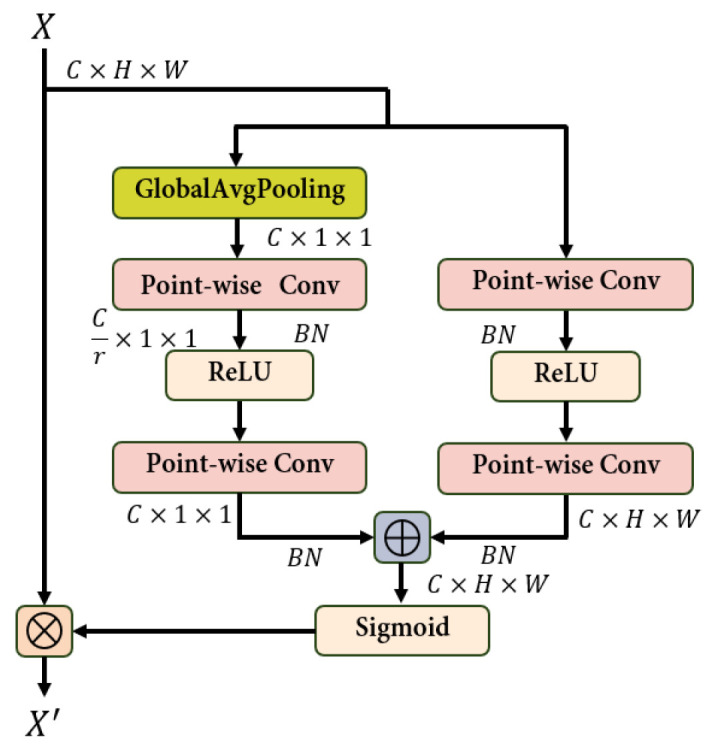
Structure of the MS-CAM.

In order to make the network pay more attention to the features of small geological bodies, the local features and global features extracted by Basic Block are input into the MS-CAM to learn the attention weights so as to aggregate the contextual semantic features of different sensitive fields of different scale targets. To keep it as lightweight as possible, only the local context is added to the global context within this module. Global channel context *G*(*X*) by the input characteristics *X* after global average pooling and local channel context $L(X)\in R^{C\times H\times W}$ by Point-wise Convolution (PWConv) are calculated as follows:

(8) $$L\left(X\right)=B(PWConv_{2}(\delta (B(PWConv_{1}(X))))),$$

where B denotes batch of normalized operation (BN), *δ* denotes ReLU linear activation operation, and $PWConv_{1},\;PWConv_{2}$ represents Point-wise Convolution. Given the global channel context *G*(*X*) and local channel context *L*(*X*), the features $X^{\prime }\in R^{C\times H\times W}$ with the number of channels *C* and size *H* × *W* can be extracted by the MS-CAM as follows:

(9) $$X^{\prime }=X⊗M\left(X\right)=X⊗\sigma \left(L\left(X\right)⊕G\left(X\right)\right),$$

where M(X) denotes attention weights generated for the MS-CAM. ⊕ denotes addition and ⊕ denotes inner product. The structure of the MS-CAM is shown in Figure 4. In MS-CAM’s aggregation of local and global context information, the local channel context *L*(*X*) has the same shape as the input feature, preserving and highlighting fine details in the underlying feature.

In the decoding process, after Basic Block and MS-CAM feature extraction four times for each level, the features of each level are upsampled to the same size, AFF is used again for attention feature fusion, and finally, the predicted model density value is output through a 1 × 1 convolution and the ReLU activation function.

Basic Block is introduced in the encoding block to keep the accuracy of the network in the deep condition and to prevent the gradient explosion or gradient disappearance in the training process. In addition, the network’s skip connection between the encoder and the decoder aims to allow the network to combine more feature information, reduce information loss in the encoding and decoding process, and help the network obtain high-resolution semantic features.

### 3.2. Loss Function

In the training process of deep neural networks, the optimization problem is usually solved by minimizing the loss function. For image regression and reconstruction problems, the L1 or L2 norm is often the most common metric used to define the loss function. However, considering that we assume that the solution of the three-dimensional gravity inversion problem has sparsity [37], that is, the part concerned by the three-dimensional gravity inversion is only a small part of the underground space, it will cause the network to converge to the local minimum in the process of gradient descent, resulting in the network prediction results biased to the background. In order to describe the edge contour of geological bodies more clearly, the Dice loss function, which can clearly describe the shape and position information of small targets even when the number of target and background voxels is unbalanced, is selected as the loss function of the inversion network [38].

Dice loss is widely used in medical image segmentation tasks to measure the similarity of two sets. The Dice coefficient is defined as follows [39]:

(10) $$DiceCoefficient=\frac{2\left|A\cap B\right|}{\left|A\right|+\left|B\right|}.$$

In this study, set *A* and set *B*, respectively, represent the set formed by the density model predicted by the network and the real density model. The dice coefficient can be specifically expressed as follows [38]:

(11) $$DiceCoefficient=\frac{2\sum_{i=1}^{N}m_{i}^{T}{\hat{m}}_{i}}{\sum_{i=1}^{N}m_{i}^{T}m_{i}+\sum_{i=1}^{N}{\hat{m}}_{i}^{T}{\hat{m}}_{i}},$$

where $m_{i}$ and ${\hat{m}}_{i}$ represent the *i*th true density model and the reconstruction of the density model, respectively, and *N* represents the total number of density models.

The loss function used in the network training process in this paper, that is, the Dice loss, is equal to the 1-Dice coefficient as follows:

(12) $$DiceLoss=1-DiceCoefficient=1-\frac{2\sum_{i=1}^{N}m_{i}^{T}{\hat{m}}_{i}}{\sum_{i=1}^{N}m_{i}^{T}m_{i}+\sum_{i=1}^{N}{\hat{m}}_{i}^{T}{\hat{m}}_{i}}.$$

The value of the Dice loss ranges from 0 to 1, and the smaller the value, the smaller the error between the network inversion results and the real label, and the better the performance of the model.

In summary, the whole inversion process can be summarized as Algorithm 1.
**Algorithm 1: A 3D gravity inversion method based on the attention fusion mechanism****Input:** Training data pair ${\left\{d\right\}}_{p=1}^{P}$, ${\left\{m\right\}}_{p=1}^{P}$, Batch size bs, Learning rate η
**Initialization:** weight $W^{\left(t\right)}$, offset $b^{\left(t\right)}$, t=0
**Repeat:** for s=1 : P//bs or P//bs+1      
$loss=0\;for\;i=1\;:\;bs_{s}$
      (1) Forward propagation      **Encoding**      
for j=1 : 4
        
${d_{j}^{i}}^{\prime }\;\;\leftarrow RELU(BN(W_{2j-1}^{\left(t\right)}*d_{j-1}^{i}+b_{2j-1}^{\left(t\right)}))$
        
$c_{j}\;\;\leftarrow RELU(BN(W_{2j}^{\left(t\right)}*{d_{j}^{i}}^{\prime }+b_{2j}^{\left(t\right)}))$
        
$d_{j}^{i}\;\;\leftarrow Downsampling\left(c_{j}\right)\;with\;Dropout(0.2)$
       
${d_{5}^{i}}^{\prime }\;\;\leftarrow RELU(BN(W_{9}^{\left(t\right)}*d_{4}^{i}+b_{9}^{\left(t\right)}))$
       
$d_{5}^{i}\;\;\leftarrow RELU(BN(W_{10}^{\left(t\right)}*{d_{5}^{i}}^{\prime }+b_{10}^{\left(t\right)}))$
       **Decoding**       
for j=4 :−1 : 1
          
${d_{j}^{i}}^{\prime }\;\;\leftarrow Upsampling\left(d_{j+1}^{i}\right)\;with\;Dropout(0.2)$
          
$a_{j}^{\prime }\;\;\leftarrow RELU(BN(W_{24-3j}^{\left(t\right)}*AFF(\;{d_{j}^{i}}^{\prime },\;c_{j}\;)+b_{24-3j}^{\left(t\right)}))$
          
$a_{j}\;\;\leftarrow RELU(BN(W_{25-3j}^{\left(t\right)}*a_{j}^{\prime }+b_{25-3j}^{\left(t\right)}))$
          
$d_{j}^{i}\;\;\leftarrow Sigmoid(MS-CAM(a_{j}))$
          ${\hat{m}}^{i}$ $\;\leftarrow RELU(AFF(d_{1}^{i},\;d_{2}^{i},\;d_{3}^{i},\;d_{4}^{i},\;d_{5}^{i}))$         
$loss\;\;\leftarrow loss+L_{i}({\hat{m}}^{i},\;m^{i})$
      (2) Back propagation         
$W^{\left(t+1\right)}\;\;\leftarrow W^{\left(t\right)}+Adam(\eta ,\;loss/{bs}_{s})$
         
$b^{\left(t+1\right)}\;\;\leftarrow b^{\left(t\right)}+Adam(\eta ,\;loss/{bs}_{s})$
**Until the neural network converges**The gravity anomaly data of the area to be reconstructed are input ***d***, and the reconstructed model $\hat{m}$ is predicted.

## 4. Experiments

### 4.1. Simulation Datasets

In the DL inversion method, in addition to designing a suitable network, it is necessary to construct a large number and various simulation data to train the network. We divided the square observation area with a surface edge length of 1600 m into 32 × 32 grids, with a sampling interval of 50 m. Divide the underground area with a length and width of 1600 m and a depth of 800 m into a rectangular prism of 32 × 32 × 16 with a side length of 50 m. Set the density of geological value to $1\;g/cm^{3}$ and the background density to $0\;g/cm^{3}$, which will be a three-dimensional inversion problem, and for the approximately 3D segmentation problem, the underground space is divided into the background region ($0\;g/cm^{3}$) and the geological area ($1\;g/cm^{3}$).

**Figure 5:**
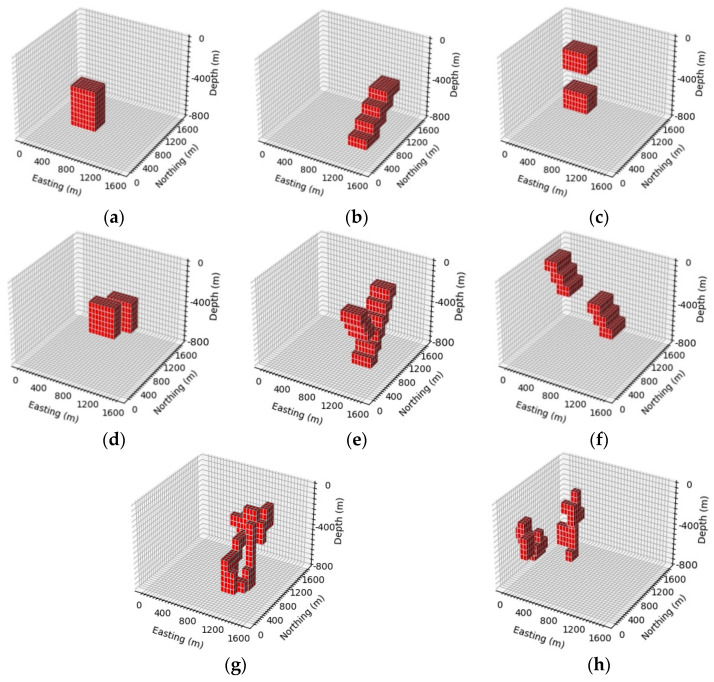
Density model of the Gravinv dataset. (**a**) Rectangular prism model, (**b**) inclined dike model, (**c**) vertical pinch-out model, (**d**) vertical parallel prism model, (**e**) syncline model, (**f**) fault model, (**g**) single random model, (**h**) combination random model.

A simulation dataset containing six conventional models was constructed, and considering the diversity of underground geologic bodies, random walk [23] was used to generate random models in order to make the network have a certain generalization ability. In this paper, a simulation dataset composed of six conventional models and random models and their corresponding abnormal data was used to train the neural network. The dataset was named the GravInv dataset. The GravInv dataset consists of a training set and a test set, with 10000 random models and 2000 for each conventional model in the training set, totaling 22000. The test set includes 100 random models and 100 for each conventional model, totaling 700 models. The six conventional and random models are shown in Figure 5.

### 4.2. Implementation Details

In the training phase, we set the number of iterations to 50 epochs, the batch size to 32, and the learning rate to 3 × 10^−4^ and adopted the Adam algorithm to optimize the network parameters (PC configuration: 11th GenIntel(R) Core(TM) i5-11320H @3.20 GHz, RAM: 16 GB; the same as below).

### 4.3. Evaluation Metrics

This paper refers to a large number of studies on 3D gravity inversion and uses the following indicators to evaluate the accuracy of the reconstruction of the gravity three-dimensional inversion model as follows:

(13) $$MAE=\frac{1}{q}\sum_{i=1}^{q}\left|\tilde{m}\left(i\right)-m(i)\right|,$$

(14) $$E_{m}=\frac{1}{q}\sum_{i=1}^{q}\frac{{\left\|\tilde{m}(i)-m(i)\right\|}_{2}}{{\left\|m(i)\right\|}_{2}},$$

(15) $$E_{acc}=\frac{C(\left|\tilde{m}(i)-m(i)\right|<t)}{q},$$

(16) $$R^{2}=1-\frac{\sum_{i=1}^{p}{\left(\tilde{d}(i)-d(i)\right)}^{2}}{\sum_{i=1}^{p}{\left(d\left(i\right)-\bar{d}\left(i\right)\right)}^{2}},$$

(17) $${\bar{E}}_{acc}=\frac{\sum_{i=1}^{w}{E_{acc}}_{i}}{w},$$

where $\tilde{m}(i)$ and m(i) represent the *i*th element in the vector of the predicted model and the vector of the theoretical model, respectively, p and q represent the dimensions of vectors d and m, respectively. The MAE is the average absolute error, reflecting the average error between the reconstructed model and the theoretical model; $E_{m}$ is the average relative error, which reflects the relative error between the real density model and the prediction model based on the L2 norm. $E_{acc}$ [31] is used to measure the inversion accuracy of the underground areas. *t* represents the threshold value; when the absolute value of the error between $\tilde{m}(i)$ and m(i) is less than the threshold value *t*, the prediction is considered correct, and 𝒞 is used to represent the number of correctly predicted models. Divide the number of correctly predicted models by the total number of outlier meshes to obtain the accuracy value. In this study, the *t* value is selected as 0.01. $R^{2}$ is a determination coefficient that reflects the degree of fitting between predicted gravity anomaly data and observed gravity anomaly data. $\tilde{d}(i)$ represents the predicted gravity anomaly value, d(i) represents the observed gravity anomaly value, and $\bar{d}(i)$ represents the mean value of observed outliers. ${\bar{E}}_{acc}$ represents the average value of $E_{acc}$ for a specific model in the test set, and w represents the number of models in the test set.

### 4.4. Simulation Datasets Experiment

#### 4.4.1. Ternary Inversion

**Figure 6:**
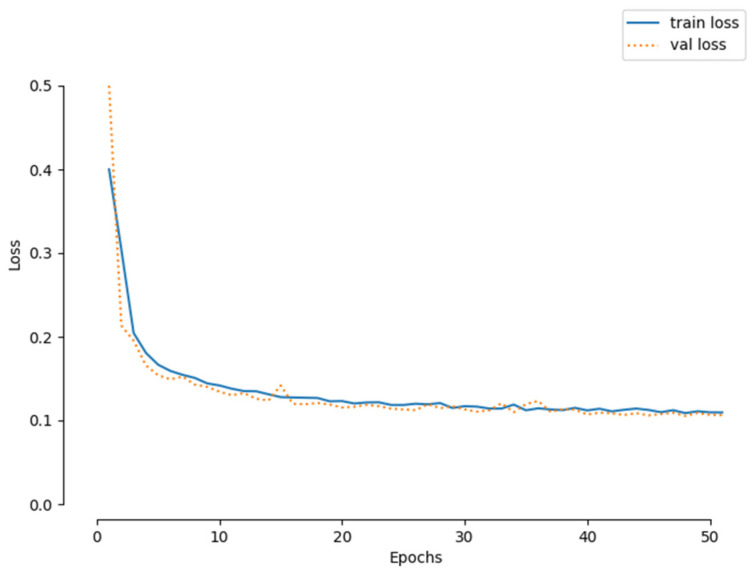
Inversion of the training loss curve of the network.

This section meticulously assesses the feasibility and validity of the proposed inversion network on the GravInv test set, ensuring a rigorous evaluation process. After undergoing 50 rounds of intensive training, the neural network successfully reconstructs the 3D density model, demonstrating its proficiency in the task. Notably, the network’s training efficiency is evident, as the loss function stabilizes around the 20th round of training, indicating a swift convergence. Figure 6 presents a compelling visualization of the loss curve for the inversion network, clearly illustrating that the training process achieves convergence within approximately 20 rounds, further validating the network’s efficiency and stability.

In order to facilitate the observation of the inversion shape of the abnormal body, all 3D views except the ablation experiment only show the part with a residual density value greater than $0.5\;g/cm^{3}$. In the following, we will select two more complex models and a random model from the test set to demonstrate the effect of 3D reconstruction of the inversion network.

**Figure 7:**
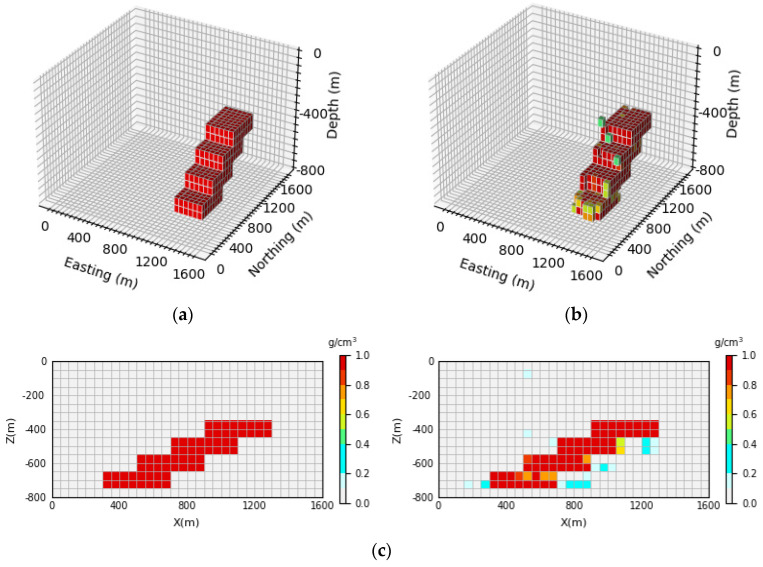
Comparison of the theoretical model and prediction model for the inclined levee model: (**a**) is the theoretical model of the verification set; (**b**) is the prediction model obtained through network inversion; (**c**) is the vertical section of the geological body, with the theoretical model section on the (**left**) and the prediction model section on the (**right**).

Figure 7 shows the 3D inversion results of the inclined levee model and the vertical section. By comparing the 3D view of the theoretical model and the predicted model, it can be observed that the network proposed in this paper cannot only well inverse the basic position and shape information of the simulated density model but also identify the buried depth range of the model. By comparing Figure 7c, it can be seen that the neural network can clearly inverse the contours and position information of the deep part of the local body, and it has a high-depth resolution. The inversion accuracy can be calculated according to Equation (15). In this paper, 100 test samples are selected for each model, and the inversion accuracy of 100 test samples is averaged to obtain the average inversion accuracy, which is expressed as ${\bar{E}}_{acc}$. After calculation, the average inversion accuracy of the model ${\bar{E}}_{acc}$ is as high as 96.43%.

**Figure 8:**
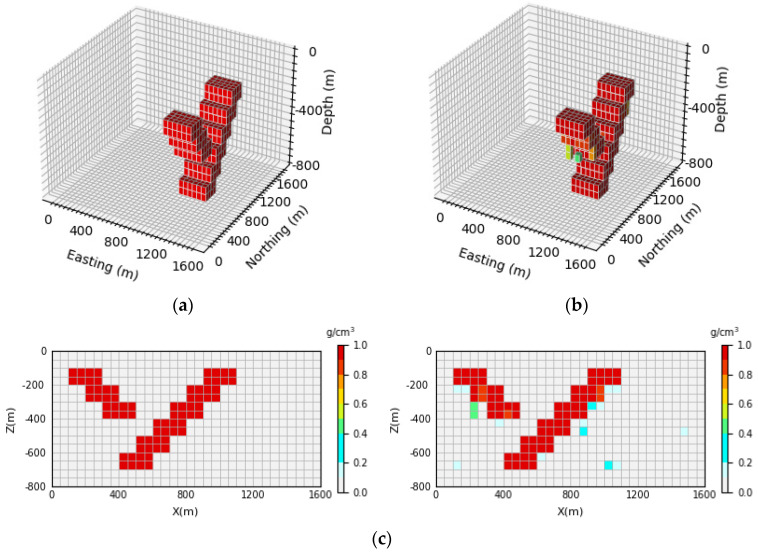
Syncline model comparison between the theoretical model and prediction model: (**a**) is the theoretical model of the verification set; (**b**) is the prediction model obtained through network inversion; (**c**) is the vertical section of the geological body, with the theoretical model section on the (**left**) and the prediction model section on the (**right**).

Figure 8 shows the 3D inversion results of the syncline model and the vertical section. By comparing the 3D view of the theoretical model and the prediction model, it can be observed that the network proposed in this paper can clearly reproduce the shape and trend of the model. By comparison with Figure 8c, it can be seen that the density model predicted by the neural network has a clear boundary in the vertical direction, which is basically consistent with the contour of the theoretical model. Moreover, the average inversion accuracy of the model ${\bar{E}}_{acc}$ is as high as 96.47%.

**Figure 9:**
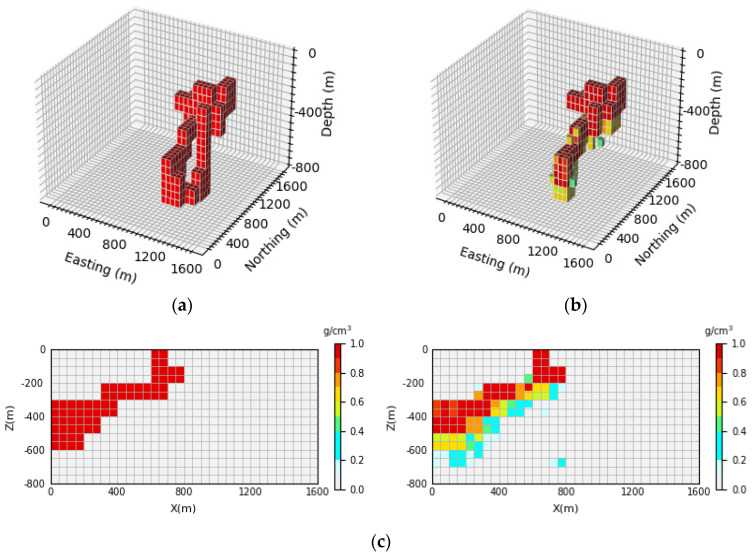
Comparison of theoretical models and predictive models for stochastic models: (**a**) is the theoretical model of the verification set; (**b**) is the prediction model obtained through network inversion; (**c**) is the vertical section of the geological body, with the theoretical model section on the (**left**) and the prediction model section on the (**right**).

Figure 9 shows the 3D inversion results of the stochastic model and the vertical section. By comparing the 3D view of the theoretical model and the prediction model, it can be observed that the network proposed in this paper still has a good inversion effect on the stochastic model and can basically reproduce the general shape and position information of the model. However, with the deepening of the depth, some information of the model is missing. By comparing Figure 9c, it can be seen that the prediction model has a relatively clear outline in the shallow part; however, with the increase in depth, the boundary of the model will inevitably become blurred, but the basic information of the density model can still be roughly distinguished. Moreover, the average inversion accuracy of the model ${\bar{E}}_{acc}$ is as high as 92.71%. It can be considered that the inversion network proposed in this paper has good generalization performance.

#### 4.4.2. Noise Experiment

**Figure 10:**
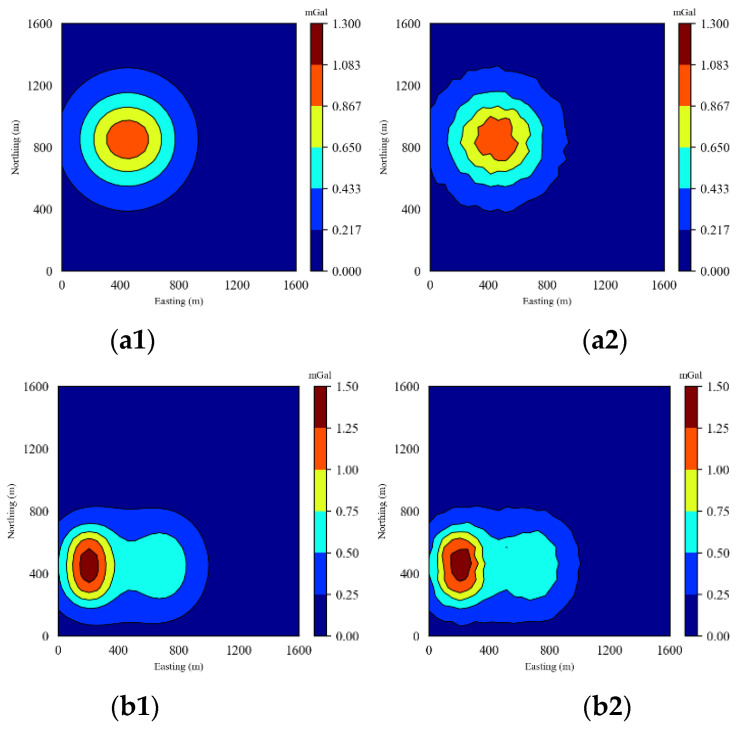
Gravity anomaly with 5% Gaussian noise added: (**a1**) is the gravity anomaly diagram of the cube model, (**a2**) is the gravity anomaly diagram of the cube model after adding 5% Gaussian noise, (**b1**) is the gravity anomaly diagram of the vertical parallel prism model, (**b2**) is the gravity anomaly diagram of the vertical parallel prism model after adding 5% Gaussian noise.

In the actual gravity anomaly inversion scenario, the gravity anomaly data obtained are often noisy. Therefore, in order to verify the robustness of the inversion network proposed in this paper, add a certain level of Gaussian noise to the gravity anomaly data constructed in Section 4.1 using noisy gravity data to train the inversion network and simulate real-case scenarios. As the noise level increases, the inversion accuracy is undoubtedly reduced. Therefore, in order to verify the robustness of the inversion network on the premise of ensuring the effectiveness of the network prediction, the method of Equation (18) is used to add noise to the gravity data [23] as follows:

(18) $$d^{nosie}=d+\lambda \times max(d)\times random(0,1,(size(d))),$$

where $d^{nosie}$ denotes the gravity data after adding noise and *λ* denotes the noise weight coefficient; to control the size of the added noise, random(0,1,(size(d))) denotes the same as the ***d*** size of Gaussian noise. In order to ensure that the added noise effectively reflects the noisy characteristics of the real data and does not overwrite the original characteristics of the gravity data, Gaussian noise with a noise weight coefficient of 5% is selected to be added to the gravity data. The gravity anomaly figure with 5% Gaussian noise added is shown in Figure 10.

**Figure 11:**
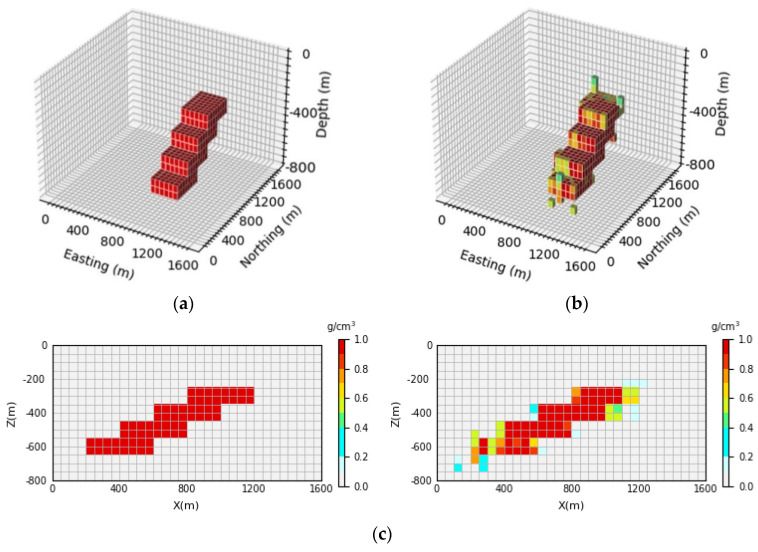
Comparison between theoretical and predictive models of inclined embankment models. (**a**) Shows the theoretical model in the validation set, (**b**) shows the prediction model obtained through network inversion trained on noisy data, and (**c**) shows the vertical profile of the geological body, with the theoretical model profile on the (**left**) and the prediction model profile on the (**right**).

**Figure 12:**
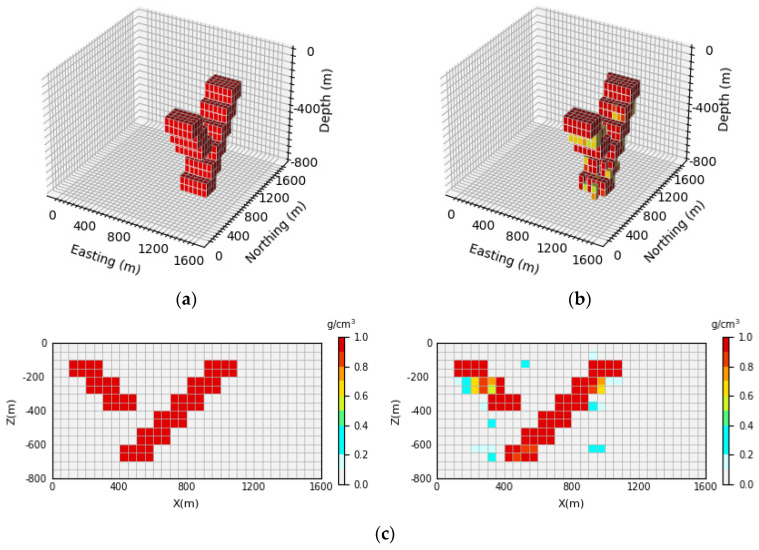
Comparison between the Diagonal Model Theory Model and the prediction model. (**a**) Shows the theoretical model in the validation set, (**b**) shows the prediction model obtained by inverting the network trained with noisy data, and (**c**) shows the vertical profile of the geological body, with the theoretical model profile on the (**left**) and the prediction model profile on the (**right**).

**Figure 13:**
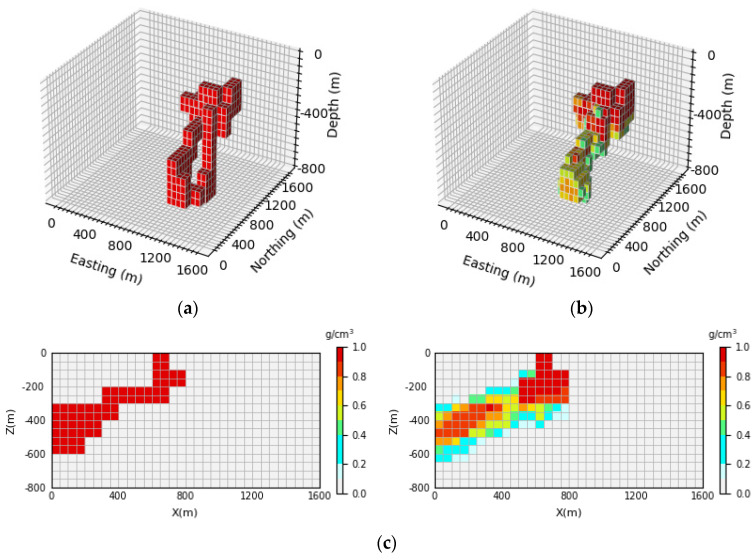
Comparison between the random model theoretical model and the prediction model. (**a**) Shows the theoretical model in the validation set, (**b**) shows the prediction model obtained through network inversion trained on noisy data, and (**c**) shows the vertical profile of the geological body, with the theoretical model profile on the (**left**) and the prediction model profile on the (**right**).

Data with added noise are used to train the inversion network, and the trained network is used to test the synthesis data without noise. The inversion results predicted by the network are shown in Figure 11, Figure 12 and Figure 13.

It can be seen in Figure 11, Figure 12 and Figure 13 that the inversion network based on the attention feature fusion mechanism has good anti-noise ability. Figure 11b, Figure 12b and Figure 13b show the 3D view of the prediction model. Both the shape and position information of the prediction model of the geological body are in good agreement with the theoretical model. It can be seen in Figure 11c, Figure 12c and Figure 13c that the prediction models all have relatively clear boundaries, which are consistent with the theoretical models in both horizontal and vertical directions. Although the boundary clarity and vertical resolution are reduced compared with the inversion results in Section 4.4.1, the average accuracy of the prediction results is still high. In the noisy experiment, the average inversion accuracy of the above three models ${\bar{E}}_{acc}$ are 94.92%, 95.07%, and 92.68%, respectively. The experimental results show that the inversion network based on the attention fusion mechanism can accurately describe the shape, location, and physical property information of the anomaly source.

The advantages of 3D gravity inversion based on DL come from the guidance of loss function and the fitting ability of neural networks to complex functions [31]. Data-driven deep learning methods determine that as more and more data are input into the neural network, its generalization and accuracy will gradually improve. The noise experiment shows that the 3D gravity inversion based on DL has better anti-noise performance, which lays a foundation for actual geological data processing.

#### 4.4.3. Contrast Experiment

**Figure 14:**
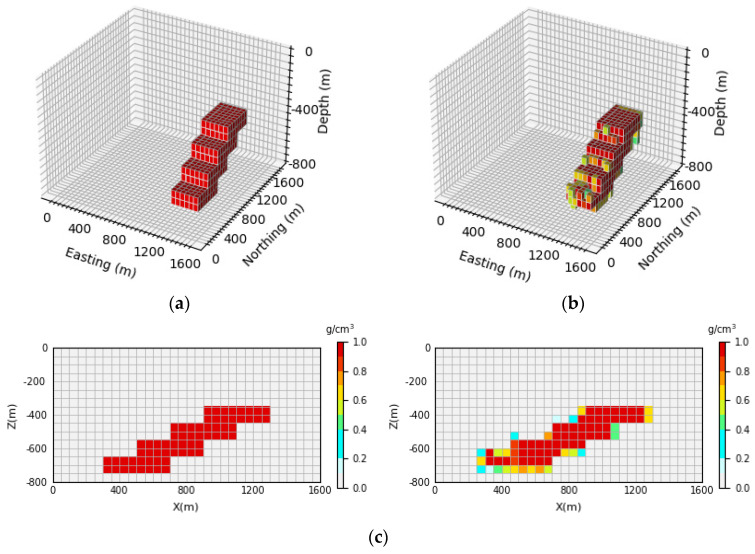
Comparison between theoretical and predictive models of inclined embankment models. (**a**) Shows the theoretical model in the validation set, (**b**) shows the prediction model obtained by U-Net inversion, and (**c**) shows the vertical profile of the geological body, with the theoretical model profile on the (**left**) and the prediction model profile on the (**right**).

**Figure 15:**
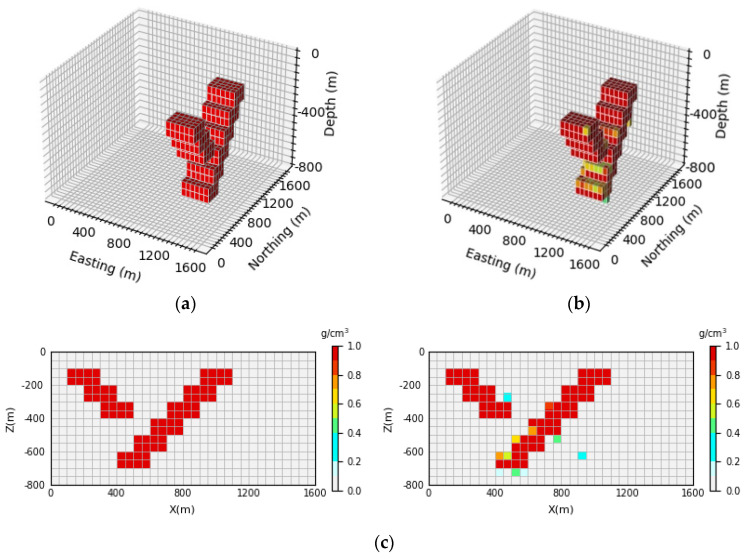
Comparison between the Diagonal Model Theory Model and the prediction model. (**a**) Shows the theoretical model in the validation set, (**b**) shows the prediction model obtained by U-Net inversion, and (**c**) shows the vertical profile of the geological body, with the theoretical model profile on the (**left**) and the prediction model profile on the (**right**).

**Figure 16:**
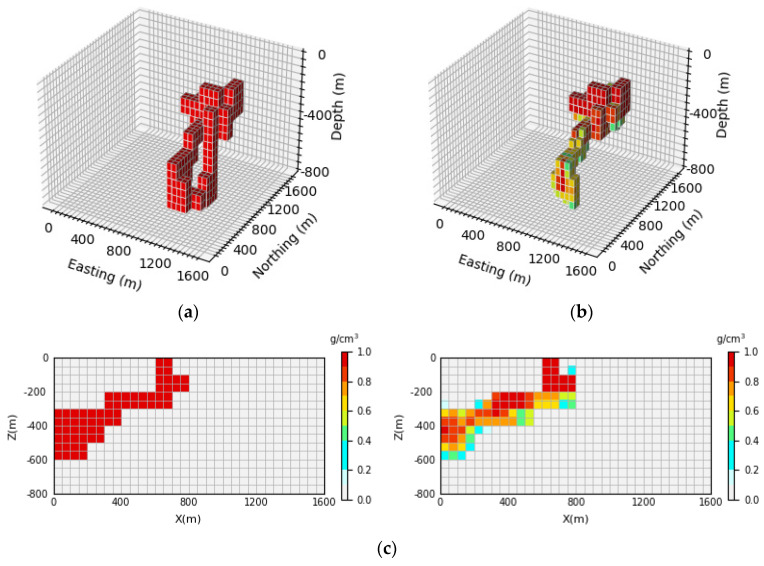
Comparison between the Diagonal Model Theory Model and the prediction model. (**a**) Shows the theoretical model in the validation set, (**b**) shows the prediction model obtained by U-Net inversion, and (**c**) shows the vertical profile of the geological body, with the theoretical model profile on the (**left**) and the prediction model profile on the (**right**).

In order to show that the inversion network proposed in this paper can alleviate the skin effect to a certain extent, the inversion results of the U-Net network are compared with those of the inversion network proposed in this subsection. The inversion results of the U-Net network are shown in Figure 14, Figure 15 and Figure 16.

As can be seen in Figure 14, Figure 15 and Figure 16, compared with the prediction model obtained by the inversion network proposed in this paper, the definition of the model boundary and the accuracy of the physical property parameters in the deeper part of the model are not as good as those in Section 4.4.1. Figure 14b, Figure 15b and Figure 16b show a 3D view of the prediction model, which is surrounded by many low-density units whose shapes are not clearly visible compared to Figure 7b, Figure 8b and Figure 9b. It can be seen in Figure 14c, Figure 15c and Figure 16c that the resolution of the prediction model gradually decreases at the deeper parts; the boundary information of the anomaly source cannot be clearly reflected, and the accuracy of physical property parameters also declines, showing a more serious skin effect. However, the inversion results in Section 4.4.1 can still clearly reverse the anomaly source boundary information in the deep part of the model, and the accuracy of the physical property parameters is also higher than the inversion results of the U-Net network. The average inversion accuracy of the three models predicted by the U-Net network ${\bar{E}}_{acc}$ is 93.51%, 94.81%, and 93.09%, respectively, which are all lower than the average inversion accuracy of the three prediction models in the inversion network in this paper. The experimental results show that the inversion network with the AFF module as the core can effectively improve the 3D inversion effect, especially in the vertical resolution, which is much better than U-Net and can alleviate the influence of the skin effect to a certain extent.

#### 4.4.4. Ablation Experiment

**Figure 17:**
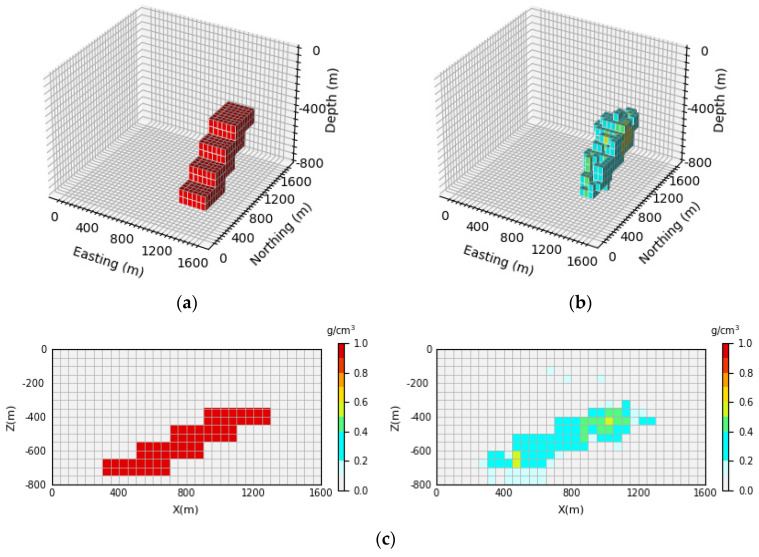
Comparison between theoretical and predictive models of inclined embankment models. (**a**) Shows the theoretical model in the validation set, (**b**) shows the prediction model obtained by removing the AFF module from the inversion network, and (**c**) shows the vertical profile of the geological body, with the theoretical model profile on the (**left**) and the prediction model profile on the (**right**).

**Figure 18:**
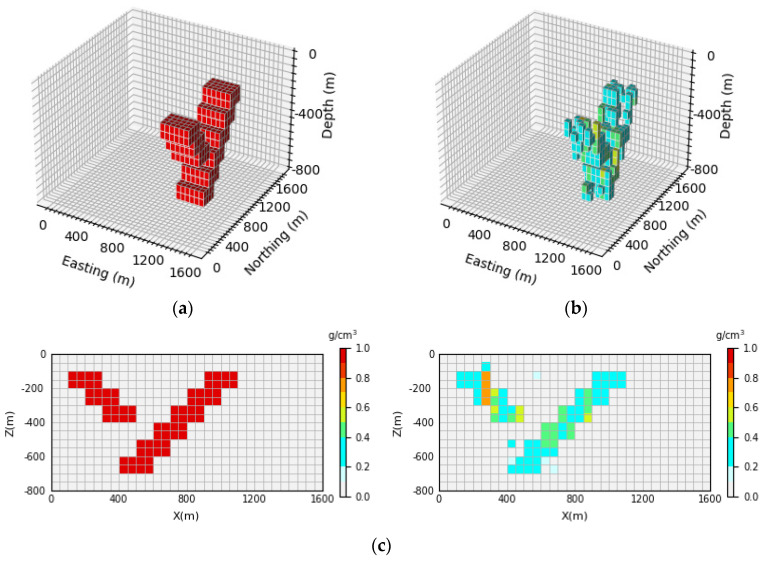
Comparison between the Diagonal Model Theory Model and the prediction model. (**a**) Shows the theoretical model in the validation set, (**b**) shows the prediction model obtained by removing the AFF module from the inversion network, (**c**) shows the vertical profile of the geological body, with the theoretical model profile on the (**left**) and the prediction model profile on the (**right**).

**Figure 19:**
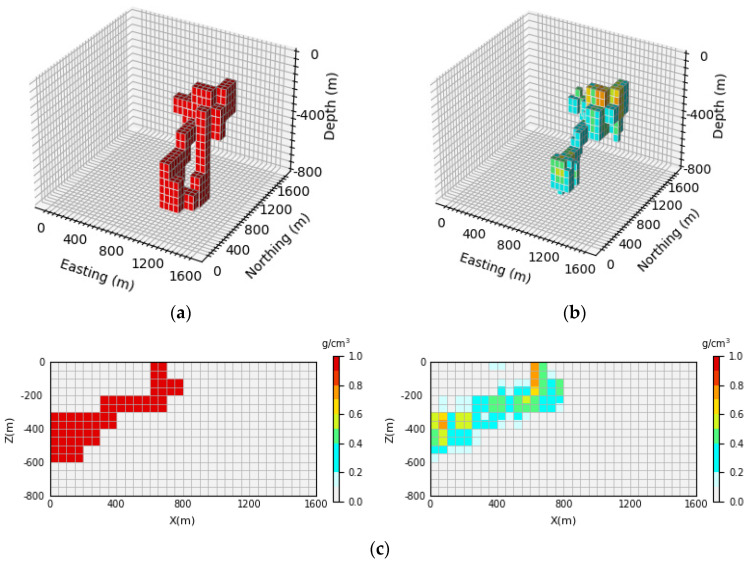
Comparison between the random model theoretical model and the prediction model. (**a**) Shows the theoretical model in the validation set, (**b**) shows the prediction model obtained by removing the AFF module from the inversion network, (**c**) shows the vertical profile of the geological body, with the theoretical model profile on the (**left**) and the prediction model profile on the (**right**).

The key point of the inversion network proposed in this paper is that the AFF module is used to aggregate the global and local context information of features on the attention channel, which reduces the possibility of losing part of the feature information in the network training process to a certain extent so that the network can learn the features of the input data more comprehensively and achieve the effect of high-precision inversion. Therefore, in order to fully demonstrate that the AFF module added in this paper can effectively improve the inversion accuracy, in this section, we will focus on comparing the inversion results without and with AFF modules in the inversion network. The GravInv dataset and the same hyperparameter setting were used to train the inversion network without the AFF module. For ease of observation, only the parts with a residual density greater than $0.3\;g/cm^{3}$ were displayed in 3D views in the ablation experiments, and the prediction results are shown in Figure 17, Figure 18 and Figure 19.

As can be seen in Figure 17, Figure 18 and Figure 19, the network prediction results are not ideal after the removal of the AFF module, which indicates that the AFF module plays a crucial role in the network proposed in this paper and is the key to high-precision 3D gravity inversion. Figure 17b, Figure 18b and Figure 19b show the 3D view of the prediction results, from which it can be seen that after the removal of the AFF module, the inversion network can only predict the general location and buried depth of the underground anomaly source, etc. However, the inversion error for the shape and physical property parameters of the anomaly source is large, which is quite different from the prediction results of the inversion network in this paper. It can be seen in Figure 17c, Figure 18c and Figure 19c that the neural network cannot accurately reproduce the shape of the anomaly source after removing the AFF module let alone obtain clear boundary information and accurate model density. Therefore, the ablation experiment shows that the AFF module is added to the inversion network in this study to learn the features of input data more effectively, which makes a great contribution to the realization of three-dimensional gravity inversion with high precision.

According to the evaluation indicators in Section 4.3, the errors of the above experiments are shown in Table 1.

In Table 1, it can be seen that the inversion network proposed in this paper occupies the smallest relative error of the model and is superior to U-Net in terms of model reconstruction error, as well as the case without the AFF module and noise. And, the inversion network proposed in this article can accurately fit surface gravity data.

The average inversion accuracy of ${\bar{E}}_{acc}$ predicted by the model is shown in Table 2. Due to the significant difference between the predicted results in the ablation experiment and the theoretical model, the experimental results are not listed here.

As can be seen in Table 2, the average inversion accuracy of the inversion network proposed in this paper is much higher than that of the other two networks for the inclined levee model and the syncline model, occupying a great advantage. The average inversion accuracy of the stochastic model is slightly lower than that of the U-Net, but the gap is not large. Compared with the other two models, the average inversion accuracy of the inversion network designed in this paper is basically negligible. Therefore, the average accuracy of the inversion network for model reconstruction is better than that of U-Net.

#### 4.4.5. Comparison of Reconstruction Effects at Different Depths

In order to more clearly observe the effect of the inversion network proposed in this article on alleviating skin effect, this section will display the xoy profile of the inversion results of the neural network at 400 m and 600 m in order to observe the effectiveness of the neural network in alleviating the skin effect.

**Figure 20:**
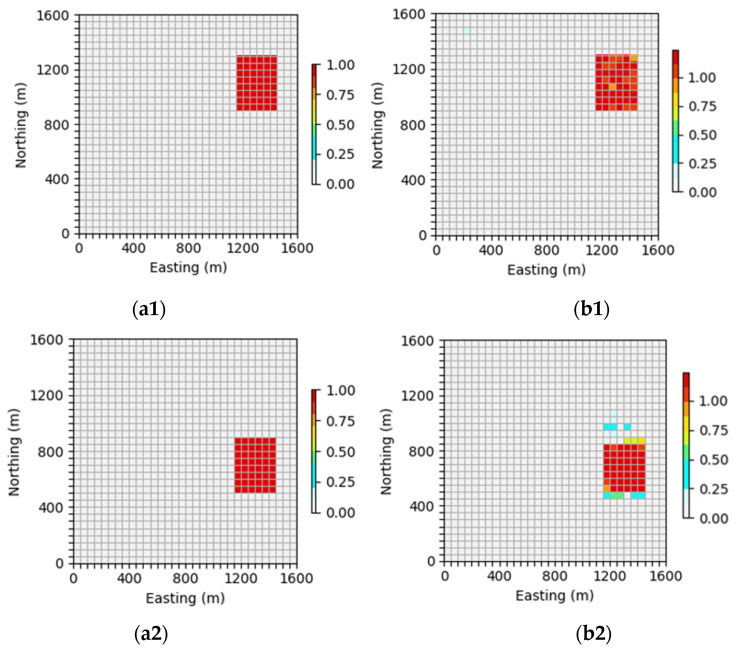
Comparison of neural network inversion profiles at different depths: (**a1**) is the profile of the inclined embankment theoretical model at 400 m and (**b1**) is the profile of the inclined embankment prediction model inverted by a neural network at 400 m; (**a2**) is the profile of the inclined embankment theoretical model at 600 m and (**b2**) is the profile of the inclined embankment prediction model inverted by a neural network at 600 m.

In Figure 20, it can be seen that the inversion network proposed in this paper can accurately invert the shape, position, and density parameters of the anomalous body when the depth is 400 m. However, when the depth is 600 m, the accuracy of the density parameters in the inversion results decreases to a certain extent. However, the predicted body shape, position, and boundary are still relatively clear, and the geological body situation can still be accurately identified.

**Figure 21:**
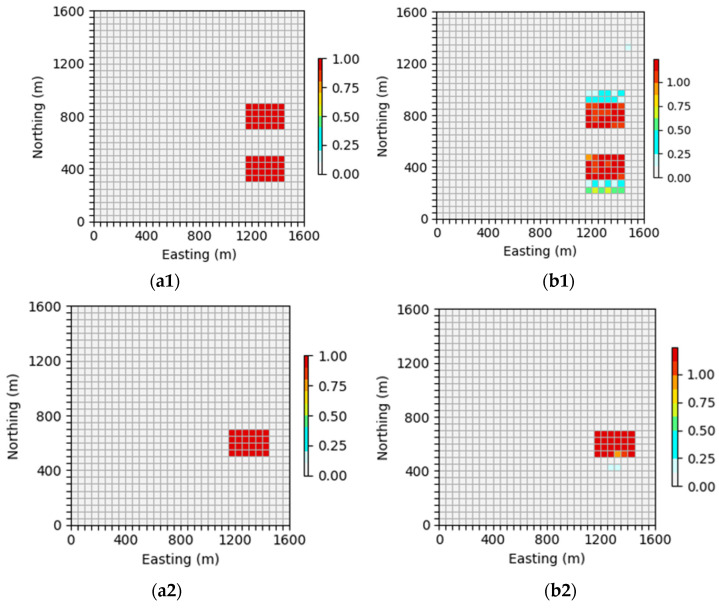
Comparison of neural network inversion profiles at different depths: (**a1**) is the profile of the theoretical model of the syncline model at 400 m and (**b1**) is the profile of the syncline model predicted by the neural network inversion at 400 m; (**a2**) is the profile of the theoretical model of the syncline at 600 m and (**b2**) is the profile of the syncline model predicted by the neural network inversion at 600 m.

As shown in Figure 21, when the depth is 400 m, the upper neural network can accurately reverse the shape, position, and density parameters of the anomalous body, but there may be some low-density impurities around the geological body. However, when the depth is 600 m, the inversion results are basically consistent with the theoretical model, and the density parameters are almost the same, indicating that the inversion network proposed in this article can better reflect the characteristics of geological bodies in deeper areas and alleviate the influence of the skin effect to a certain extent.

**Figure 22:**
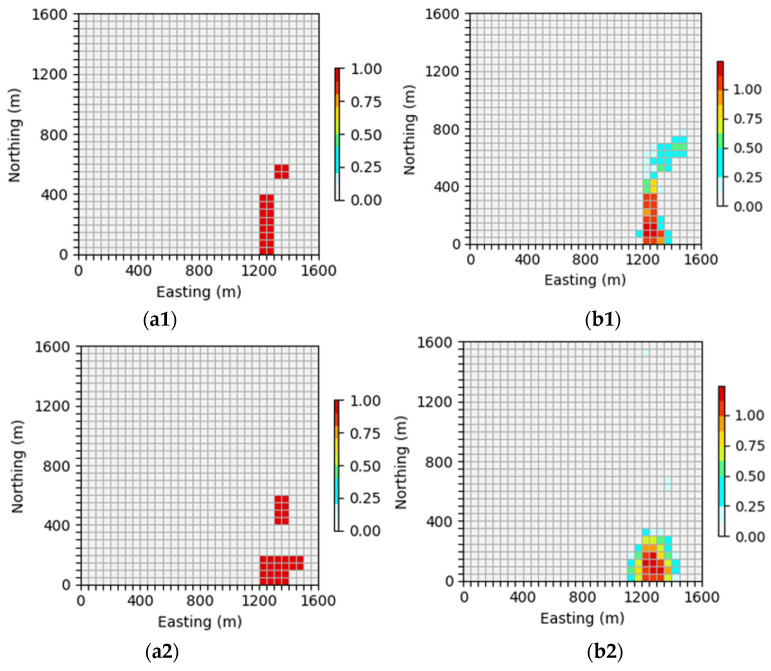
Comparison of neural network inversion profiles at different depths: (**a1**) is the profile of the random model theoretical model at 400 m and (**b1**) is the profile of the neural network inversion random model prediction model at 400 m; (**a2**) is the profile of the random model theoretical model at 600 m and (**b2**) is the profile of the neural network inversion random model prediction model at 600 m.

In Figure 22, it can be seen that when the depth is 400 m, the neural network can basically invert the shape and position of the anomalous body, and the density parameters of the inversion results are also close to the theoretical model. When the depth is 600 m, the neural network can accurately deduce the location information of the abnormal body and roughly deduce the shape of the abnormal body, and the physical boundary is relatively fuzzy. There is a certain gap between the prediction of the density parameters and theoretical models.

In summary, a comprehensive comparison of the inversion results across various depths of the aforementioned models reveals a distinct advantage of the inversion network introduced in this paper. Specifically, it is evident that this network significantly enhances the reconstruction accuracy for deep models, showcasing its superiority over traditional methods. Furthermore, it effectively mitigates the impact of the skin effect to a notable extent, contributing to more accurate and reliable inversions.

### 4.5. Field Example

In order to verify that the inversion network designed in this paper is meaningful in processing actual data, the inversion network is applied to the actual gravity anomaly data in the field of the SAN Nicolas deposit.

**Figure 23:**
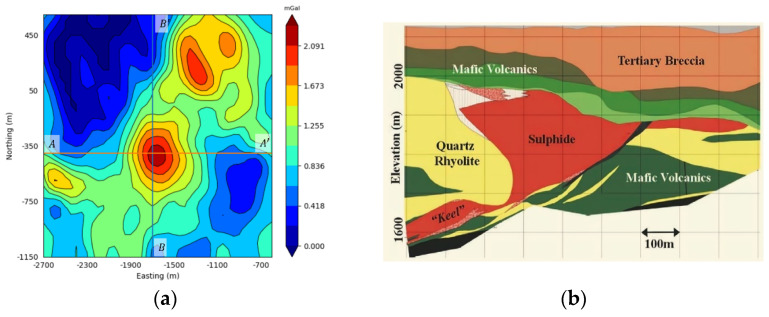
Residual gravity anomaly map and geological section: (**a**) residual gravity anomaly map of the SAN Nicholas deposit, including *AA*’ and *BB*’, which represent two drilling lines, and (**b**) is the *AA*’ line drilling geological section [40].

The San Nicholas deposit in central Mexico is an unexploited massive sulfide deposit formed by volcanic eruptions [40], occurring in the feldspathic magnesian volcanic rock sequence from the Upper Jurassic to the Lower Cretaceous, containing gold, silver, copper, zinc, and other substances [41]. Drilling data show that the top of the sulfide ore body is located 150–220 m below the surface, and the bottom of the ore body extends 400–450 m below the surface, with a thickness of about 280 m [41]. The residual gravity anomaly map of the study area is shown in Figure 23. The gravity anomaly in the center of the region is caused by massive sulfides, while other anomalies are caused by ferromagnetic volcanic rocks. The residual gravity anomaly can reach up to 2.2 mGal. As can be seen in Table 3, the density value of the sulfide is 3.5 $g/cm^{3}$, and the density value of the surrounding rock is distributed between 2.3 $g/cm^{3}$ and 2.7 $g/cm^{3}$ [42]. Therefore, the residual density value of the study area is 1.1 $g/cm^{3}$.

**Figure 24:**
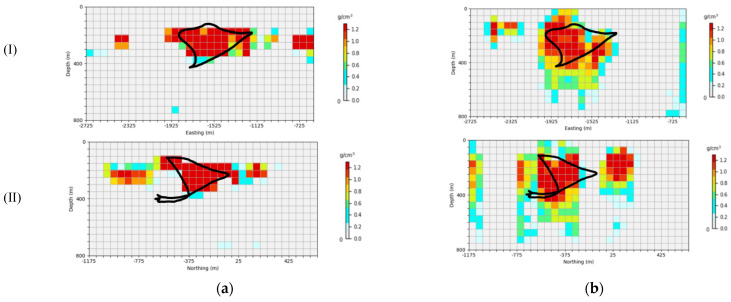
Inversion profile of the SAN Nicholas deposit: (**a**) prediction results of the inversion network in this paper, (**b**) prediction results of U-Net, (Ⅰ) Easting=−1700 m, (Ⅱ) Northing=−400 m. The black line is the actual outline of the ore body.

The density prior information was set as 1.1 $g/cm^{3}$. In order to enable the inversion network to more accurately reflect the shape, location, and physical property parameters of underground sulfide in the SAN Nicholas mining area, a dataset with a model density of 1.1 $g/cm^{3}$ was re-generated to train the inversion network. Figure 24 shows the retraining of the inversion network to the Saint Nicholas prediction results of the measured data in the deposit, Figure 24a and Figure 24b are, respectively, the $AA^{\prime }$ line in Northing=−400 m and $BB^{\prime }$ in Easting=−1700 m vertical profiles, and the black line represents the actual contour ore bodies. It can be seen in Figure 24 that the predicted distribution of the inversion network proposed in this paper is focused, the position information of the predicted results is basically consistent with the drilling information, and the abnormal source shape and physical boundary of the predicted model are also consistent with the actual situation. However, although the position information of U-Net is consistent with the actual situation, the inversion of the physical boundary is relatively fuzzy; especially, when the depth deepens, the resolution of the prediction results begins to decline, and the prediction at the bottom of the anomaly source is quite different from the actual situation. Therefore, in the scenario of the SAN Nicholas actual address data, the inversion network proposed in this paper has a more significant advantage in predicting deep anomalies.

## 5. Conclusions

In this paper, we propose a 3D gravity inversion method based on U-Net using an attentional feature fusion mechanism. With U-Net as the basic network framework, the residual module is used to replace the convolutional layer, and the operation of splicing features after jumping connections in U-Net is replaced by the fusion of contextual semantic features through the attention feature fusion module so as to enable the network to fully learn the features of the input data and avoid the feature loss in the network training process. The Dice loss function, which can clearly describe the shape and position information of small targets, even when the number of target and background voxels is unbalanced, is used as the loss function. Compared with the inversion results of the U-Net network, the method proposed in this paper has a higher vertical resolution. Ablation experiments show that the attention feature fusion module added to the network in this paper is the key to improving the vertical resolution and prediction accuracy of the inversion results. Meanwhile, noise experiments show that the inversion network in this study has a strong anti-noise ability and good generalization. It also lays a foundation for the inversion network in this paper to process the actual address data. The experimental results of the inversion network used in the prediction of the SAN Nicolas deposit in Mexico show that the inversion network can basically delineate the basic position and shape of the sulfur deposit, and the network has certain generalization performance, which is a promising subsurface density estimation tool.

## Figures and Tables

**Table 1:** Comparison of prediction errors of the U-Net, inversion network, noise experiment, and ablation experiment.

	U-Net	Our Method (W/O AFF)	Our Method (Add Noise)	Our Method
MAE	0.0074	0.0175	0.0114	**0.0072**
E_m_	0.3648	1.9173	0.4649	**0.3605**
R^2^	0.9447	0.2859	0.9162	**0.9813**

The bold font in the table represents the numerical values of the evaluation indicators corresponding to the model with the best reproduction effect.

**Table 2:** ${\bar{E}}_{acc}$ of the U-Net, inversion network, and noise-added experiment.

	U-Net	Our Method (Add Noise)	Our Method
Inclined levee model	93.51%	94.92%	**96.43%**
Syncline model	94.81%	95.07%	**96.47%**
Stochastic model	**93.09%**	92.68%	92.71%

The bold font in the table represents the numerical values of the evaluation indicators corresponding to the model with the best reproduction effect.

**Table 3:** Physical properties of major rock units in the SAN Nicolas deposit.

Rock Type	$\mathrm{Density}\;(g/cm^{3}$)
Tertiary breccia	2.3
Mafic volcanics	2.7
Sulphide	3.5
Quartz rhyolite	2.4
Graphitic mudstone	2.1

## Data Availability

The data underlying this paper cannot be shared publicly. The data will be shared upon reasonable request to the corresponding author.
